# Association of dietary patterns of pregnant women with pregnancy outcomes: A hospital‐based study

**DOI:** 10.1002/fsn3.3726

**Published:** 2023-10-03

**Authors:** Sahar Ghorbani‐Kafteroodi, Maryam Ghiasvand, Maryam Saghafi‐Asl, Soudabeh Kazemi Aski

**Affiliations:** ^1^ Student Research Committee Tabriz University of Medical Sciences Tabriz Iran; ^2^ School of Nutrition and Food Sciences Tabriz University of Medical Sciences Tabriz Iran; ^3^ Nutrition Research Center, Department of Clinical Nutrition, School of Nutrition and Food Sciences Tabriz University of Medical Sciences Tabriz Iran; ^4^ Reproductive Health Research Center, Department of Obstetrics and Gynecology, Al‐Zahra Hospital, School of Medicine Guilan University of Medical Sciences Rasht Iran

**Keywords:** anthropometric, Apgar score, birth weight, dietary patterns, gestational weight gain, pregnancy

## Abstract

Diet is one of the main factors influencing pregnancy outcomes. Maternal and child health both seem to be related to dietary patterns. So far, no study on dietary pattern has been performed on pregnant women and its association with pregnancy outcomes in Rasht. Therefore, the present study aimed to investigate the association between dietary patterns and pregnancy outcomes in Rasht. In this cross‐sectional study, 300 healthy pregnant women were included from three public hospitals in Rasht. Data on demographic, dietary intake, physical activity (PA), and anthropometric measurements of mothers were recorded. Outcomes of newborns were also gathered. Dietary patterns were identified using principal component analysis. General linear model was used for data analysis. Prior to pregnancy, only 40% of women had a normal body mass index (BMI). More than half of them (52.3%) had a gestational weight gain in excess of the guidelines. The dominant dietary patterns among pregnant women were traditional, Western, and healthy, respectively. High adherence to the Western pattern had a direct association with gestational weight gain (*B* = 1.48, *p* = .046) and inverse association with birth length (*B* = −0.71, *p* = .043). However, the results did not remain significant after adjusting for covariates. The present study indicated that several factors can affect the association of the Western diet with pregnancy outcomes. Therefore, making policies for interventional programs to improve maternal lifestyle factors along with their diet quality is recommended.

## Graphical abstract

This cross‐sectional study indicated that only in crude model, higher adherence to Western dietary pattern resulted in higher gestational weight gain and reduced birth length. There was no significant association between the Western dietary pattern and baby's weight and head circumference. There was no significant association between healthy eating patterns and pregnancy outcomes. There was no association between traditional dietary pattern and pregnancy outcomes.

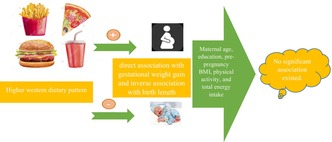

## 1 INTRODUCTION

Pregnancy is a critical period for the offspring's metabolic development (Barker et al., 2008). Dietary patterns prior to and during pregnancy have implications for the health of both mother and baby (Jiang et al., 2019). Over the past decade, studies have demonstrated that besides genetic potential, environmental factors, placenta structure, maternal diets, and nutritional status during pregnancy could influence birth outcomes directly or/and indirectly in the fetus (Chen et al., 2018; Johnston et al., 2002; Krishna & Bhalerao, 2011; McCullough et al., 2017). During pregnancy, sufficient nutrients should be provided for adequate fetal growth (Ma et al., 2019).

In adulthood, some chronic diseases originate from early nutritional status, which is named ‘nutrition programming’ (Godfrey & Robinson, 1998). The “Barker hypothesis” proposed in 1990 by the British epidemiologist David Barker (1938–2013) posits that, in humans, intrauterine growth retardation, low birth weight (LBW), and premature birth have a causal relation to the origin of hypertension, cardiovascular disease, and type 2 diabetes in middle‐aged adults (Barker, 1997; Godfrey & Barker, 2001; Kind et al., 2006). Therefore, changes in utero can have negative impact on the neonate's health later in life. For example, it can increase the risk of cardiovascular diseases and diabetes (Li et al., 2015), according to the Developmental Origins of Health and Disease (DOHaD) policies (Barker et al., 2002; Fleming et al., 2015; Gage et al., 2016).

While several studies on maternal diet have focused on the specific nutrients or individual foods in relation to birth outcomes, the associations between total diet quality and birth outcomes are not well understood (Englund‐Ögge et al., 2019; Grieger & Clifton, 2014; Mitchell et al., 2004). Although each individual food item is consumed as part of a total, the influence of a single nutrient might be difficult to ascertain (Englund‐Ögge et al., 2019). Therefore, in recent years, the focus of research has shifted toward analyzing dietary patterns (Englund‐Ögge et al., 2019).

It is noteworthy that available data about the association of maternal dietary patterns and birth outcomes such as neonatal anthropometric measurements are limited in the Middle East where they differ from Western countries (Rafieifar et al., 2016). However, anthropometric measures, consisting of birth weight, length, and head circumference, are extensively assessed as determinants of growth adequacy in fetal and maternal nutrition (Zhang & Li, 2015). Inadequate fetal growth especially in head circumference is correlated with nonoptimal neurodevelopmental outcomes (Sicard et al., 2017). Birth weight is one of the important indicators of child survival and reveals the risk of hazards to health and death in the first year of life (Coelho et al., 2015). Gestational weight gain (GWG) is also established as one of the predictors of fetal growth and pregnancy outcomes (Li et al., 2013; Wen & Lv, 2015).

Several studies have attempted to establish associations between maternal dietary patterns and perinatal outcomes; however, results are inconsistent (Abdollahi et al., 2021). For instance, Englund‐Ögge et al. (2019) in a Norwegian cohort study on dietary patterns among Norwegian mothers reported that compared with the high Western pattern, the high prudent pattern was associated with lower birth weight and the high traditional pattern with higher birth weight for all three growth standards (Englund‐Ögge et al., 2019). A study (Hajianfar et al., 2018) on maternal dietary patterns during early pregnancy and their association with neonatal anthropometric measurements found a positive significant association between high adherence to Western dietary pattern and chance of having a low‐birth‐weight infant, whereas such associations were not observed in women taking healthy or traditional dietary patterns (Hajianfar et al., 2018). The other study (Ancira‐Moreno et al., 2020) on dietary patterns and diet quality and birth outcomes in Mexico demonstrated that there were no associations between dietary patterns (healthy and mixed) and low birth weight risk (Ancira‐Moreno et al., 2020). Wrottesley et al. (2017) in South Africa showed the influence of maternal dietary pattern on GWG in urban black women. They demonstrated that Western and mixed dietary patterns were associated with higher GWG in normal‐weight and obese women. Additionally, having a traditional dietary pattern was associated with reduced odds of excessive weight gain in the total, as well as normal‐weight women (Wrottesley et al., 2017). A cross‐sectional study (Zuccolotto et al., 2019) in Brazilian pregnant women showed that mothers who consumed healthier and Brazilian traditional pattern had lower chance of obesity, compared to women who consumed lower healthy and Brazilian traditional pattern (Zuccolotto et al., 2019). However, Saldiva et al. (2022) showed that although Brazilian traditional pattern had a defensive effect on GWG, women who were obese at the start of the study had higher chances of excessive GWG (Saldiva et al., 2022). There are very few studies (Englund‐Ögge et al., 2019; Hajianfar et al., 2018; Saldiva et al., 2022; Wrottesley et al., 2017; Zuccolotto et al., 2019) examining the association between dietary patterns and pregnancy outcomes with inconsistent results. In Iran, very limited studies have been conducted on neonatal anthropometric measurement and GWG which include those in the Center and South‐West regions (Angali et al., 2020; Hajianfar et al., 2018). Moreover, no studies have examined dietary patterns and pregnancy outcomes in Rasht. Therefore, considering the increasing prevalence of unfavorable pregnancy outcomes with insufficient nutritional status of pregnant mothers (Abu‐Saad & Fraser, 2010; Black et al., 2008; Kramer, 2003) and that dietary patterns differ between countries and populations (Hu, 2002), the present study was carried out to examine the association of maternal dietary patterns with pregnancy and birth outcomes among pregnant women in Northern Iran.

## 2 METHODS

### 2.1 Study population and setting

The current cross‐sectional study was conducted from February 2021 to January 2022 in Rasht, Guilan, Iran. Three‐hundred apparently healthy pregnant women, referred to three public hospitals in Rasht, were selected by simple random sampling method in proportion to the number of referrals of pregnant mothers for delivery. Inclusion criteria were as follows: living in Guilan province, consent to participate in the study, age 18–45 years, term pregnancy (after 37 weeks), single fetus, natural vaginal delivery and cesarean section, normal pregnancy and IVF, and no smoking. Unwillingness to participate in the study, having any gynecological disorders and diseases caused by pregnancy complications (infection, gestational hypertension, preeclampsia, eclampsia, and gestational diabetes), food allergy, emergency delivery, or history of COVID‐19 were regarded as exclusion criteria. In addition, those on a special diet (e.g., to gain more weight, etc.) or taking certain supplements (weight gain supplements) and medications (psychiatric medicine, epilepsy and seizure medicine, or addiction medicine) that would affect the weight of mother and baby were excluded from the study. Informed consent was obtained from all participants. The study was approved by the ethics committee of Tabriz University of Medical Sciences, Tabriz, Iran (Ethical code: TBZMED.REC.1400.220).

### 2.2 General characteristics and anthropometrics assessments

General characteristics of the study women including age, gestational age, education, job, midwifery information (number of pregnancies, number of deliveries, history of abortion, stillbirth), and history of diseases (at prepregnancy, previous pregnancy, and current pregnancy) were obtained via a checklist. Physical activity (PA) data were attained through short‐form International Physical Activity Questionnaire (SF‐IPAQ) (Sanda et al., 2017).

Pregnant mothers' weight was measured by light clothing without shoes and their height was measured by a gauge in a standing position before delivery. Prepregnancy weight was extracted from a pregnancy booklet provided by the health center or the mother's medical record. In the absence of relevant data, the pregnant mother was asked to self‐report which was then rechecked through the health center where they referred during pregnancy.

Body mass index (BMI) was obtained by dividing weight in kilograms by height squared in meters and weight gain was calculated from the difference between prenatal weight and prepregnancy weight. Neonatal anthropometrics including height, weight, head circumference, and Apgar score were also extracted from the medical records after birth.

### 2.3 Dietary assessment

To evaluate food intake, a validated semiquantitative food frequency questionnaire (FFQ) of 147 food items was used (Asghari et al., 2012). It should be noted that the FFQ was related to the frequency of food consumption during the third trimester of pregnancy, the relevant data were collected immediately after the mother was recovered in the hospital (before discharge). The frequency of each food consumption during the last trimester of pregnancy was reported as daily, weekly, or monthly by the individuals. Then, food intake data were reported on a daily basis as grams.

### 2.4 Statistical analysis

To extract dietary patterns, first, the 147‐item FFQ was categorized into 23 food groups according to the food similarity. Then, factor analysis (i.e., principal component analysis (PCA)) was created with orthogonal varimax rotation. Dietary patterns were determined by scree plot, eigenvalues >1.5, and factor interpretation. Factor loadings ≥0.2 were considered to determine food groups related to food patterns. The scores of mothers' food patterns were categorized into tertiles, as below: Tertile 1 (low score), Tertile 2 (medium score), and Tertile 3 (high score). Tertile 1 of each food pattern was considered as a reference.

To express general characteristics, mean (SD) for continuous variables and frequency (%) for categorical variables were used. ANOVA test was performed to investigate significant differences in energy and nutrient intake of the pregnant women among tertiles of dietary patterns. General linear model (GLM) was used to assess the association between dietary patterns and pregnancy outcomes (GWG, weight, height, and head circumference of newborns). The role of the confounders including age, education, prepregnancy BMI, physical activity, and energy intake were also considered. SPSS software (Version 25, SPSS, Inc.) was applied for all statistical analyses.

## 3 RESULTS

### 3.1 Characteristics of study population

**FIGURE 1:**
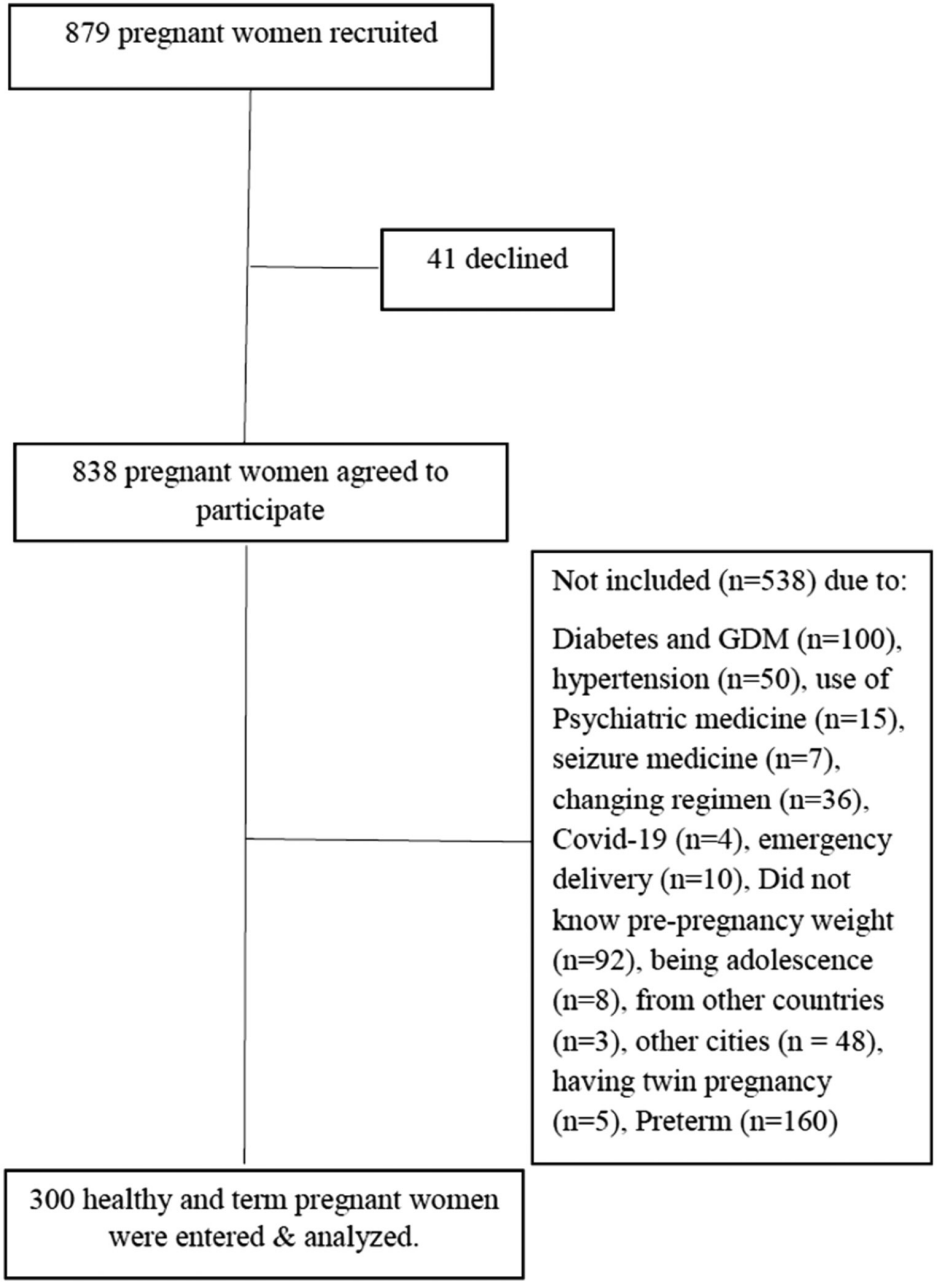
Flow chart of the study participants.

In total, 300 pregnant women in the age range of 18–45 years (mean = 29 years) who completed term pregnancy (the mean gestational age: 38.36 weeks) were entered into the study (Figure 1). Most of the participants had diploma education (41.3%), housework (93.7%), and low physical activity (80.3%). Prior to pregnancy, nearly 40% (39.7%) of women had a normal body mass index (BMI), with nearly 32% and 25% of the participants considered as overweight or obese. More than half of them (52.3%) had a weight gain in excess of the IOM gestational weight gain guidelines during pregnancy (Table 1).

**TABLE 1:** General characteristics of the study participants in North of Iran (*n* = 300).

Variables	Mean (SD)
Maternal age at delivery (years)	29 (5.9)
Gestational age (weeks)	38.36 (0.94)
Weight before pregnancy (Kg)	69.07 (15.28)
Gestational weight gain (Kg)	13.51 (5.31)

Variables	*n* (%)
Prepregnancy BMI	
Underweight	13 (4.3)
Normal	119 (39.7)
Overweight	94 (31.3)
Obese	74 (24.7)
Adequacy of gestational weight gain	
Insufficient	48 (16)
Adequate	95 (31.7)
Excessive	157 (52.3)
Occupation	
Housewife	281 (93.7)
Self‐employed	12 (4)
Employee	7 (2.3)
Physical activity	
Sedentary/low	241 (80.3)
Moderate/high	59 (19.7)
Education	
Under diploma	114 (38)
Diploma	124 (41.3)
Higher	62 (20.7)
History of miscarriage	
0	243 (81)
1	42 (14)
≥2	15 (5)
History of diseases	
None	272 (90.7)
Urinary tract infections	4 (1.3)
Anemia	8 (2.7)
Multiple sclerosis	1 (3)
Hypothyroidism	15 (5)
Previous live births	
0	140 (46.7)
1	129 (43)
≥2	31 (10.3)
Fetal sex	
Female	147 (49)
Male	153 (51)

Abbreviations: BMI, body mass index; SD, standard deviation.

### 3.2 Neonatal anthropometric parameters

Average weight, height, and head circumference of the baby were 3309.90 (439.65) kg, 49.69 (2.52) cm, and 34.62 (1.31) cm, respectively. Average 1‐min Apgar score and 5‐min Apgar score were above seven in all newborns (Table 2).

**TABLE 2:** Birth outcomes of the study participants (*n* = 300).

Variables	Mean (SD)
Birthweight	3309.90 (439.65)
Birth length	49.69 (2.52)
Birth head circumference	34.62 (1.31)
1‐min Apgar score	8.39 (0.53)
5‐min Apgar score	9.40 (0.53)

*Note*: Values have been presented as Means ± SD.

### 3.3 Dietary patterns

Using the factor analysis method, three dominant dietary patterns were obtained, which were labeled as traditional, Western, and healthy according to factor loadings and food groups (Table 3). The traditional pattern showed high positive factor loadings for legumes, sweets, potatoes, tea and coffee, bread and grain, red and processed meat, butter, soft drinks, and high‐fat dairy products. The Western pattern had high positive factor loadings for pizza, snacks, spices, visceral meat, nuts, olive, mayonnaise, industrial juices, and compote and negative factor loading for tea and coffee. The healthy pattern was characterized by positive factor loadings for liquid oil, vegetables, fruits, and low‐fat dairy products, as well as negative factor loading for solid oil. These three dietary patterns explained 26.37% of the total diet variance.

**TABLE 3:** Dietary patterns derived from principal component analysis and their corresponding coefficients identified in pregnant women in North of Iran.

Food groups	Dietary pattern		
	Traditional	Western	Healthy
Pizza	‐	0.649	‐
Snacks	‐	0.571	‐
Spices	‐	0.551	‐
Visceral meat	‐	0.487	‐
Nuts	‐	0.476	‐
Olive	‐	0.472	‐
Mayonnaise	‐	0.368	‐
Compote and industrial juices	‐	0.282	‐
Poultry	‐	‐	‐
Legumes	0.546	‐	‐
Sweets	0.500	‐	‐
Potato	0.482	‐	‐
Tea and Coffee	0.427	−0.243	‐
Bread and Grains	0.382	‐	‐
Red and processed meats	0.350	‐	‐
Butter	0.329	‐	‐
Soft drinks	0.300	‐	‐
High‐fat dairy	0.296	‐	‐
Solid oil	‐	‐	−0.761
Liquid oil	‐	‐	0.730
Vegetables	‐	‐	0.411
Fruit	‐	‐	0.361
Low‐fat dairy	‐	‐	0.305

*Note*: Values less than 0.20 have been omitted.

### 3.4 Energy and macronutrient intake under different dietary patterns

The average intake of energy and macronutrients of the studied pregnant women according to the score of food patterns are shown in Table 4. The intake of energy and macronutrients in the traditional pattern was significantly higher than that in the Western and healthy pattern (*p* < .001; Table 4).

**TABLE 4:** Energy and macronutrient intake in pregnant women based on dietary patterns.

Energy and macronutrients	Dietary patterns			*p* Value
	Traditional	Western	Healthy	
Energy (kcal/day)	2844.77 (529.95)	2588.90 (704.81)	2366.39 (567.43)	>0.001
Carbohydrates (g/day)	415.08 (87.43)	360.93 (97.51)	338.29 (87.94)	>0.001
Protein (g/day)	97.80 (23.39)	90.55 (30.06)	83.004 (26.36)	>0.001
Fat (g/day)	95.84 (24.92)	94.87 (30.95)	83.02 (23.05)	>0.001
SFA (g/day)	29.26 (10.13)	25.56 (9.73)	22.34 (8.82)	>0.001
Fiber (g/day)	49.33 (21.63)	38.70 (15.06)	38.27 (14.78)	>0.001

*Note*: Values have been presented as Means ± SD. *p* Values based on ANOVA (analysis of variance).

Abbreviations: SD, standard deviation; SFA, saturated fatty acid.

### 3.5 Association between dietary patterns and pregnancy outcomes

Results of the general linear model (GLM) analysis showed that there was no statistically significant association between traditional dietary pattern and mother's weight gain, baby's birth weight, baby's height, and baby's head circumference. Also, after adjusting for covariates such as age, education level, prepregnancy BMI, physical activity, as well as energy intake, this association remained unaltered. Women in the highest tertile of the Western dietary pattern gained 1.48 kg more weight (*B* = 1.48, *p* = .046) and the baby's height was 0.71 cm shorter (*B* = −0.71, *p* = .043) compared to the lowest tertile. However, the results did not remain significant after adjusting for the covariates. There was no significant association between the Western dietary pattern and baby's weight and head circumference. Statistical analyses did not show a significant association between healthy eating patterns and pregnancy outcomes (Table 5).

**TABLE 5:** Association between different dietary patterns and pregnancy outcomes.

Outcomes	Unadjusted				Adjusted^a^			
	Tertile 2		Tertile 3		Tertile 2		Tertile 3	
	*B* (SE)	95% CI	*B* (SE)	95% CI	*B* (SE)	95% CI	*B* (SE)	95% CI
Traditional dietary pattern								
Gestational weight gain (Kg)	1.13 (0.74)	−0.33, 2.59	0.79 (0.74)	−0.67, 2.55	1.21 (0.77)	−0.31, 2.73	0.13 (0.98)	−1.80, 2.07
Tertile 1 is selected as *reference* group.Birth weight (g)	7.70 (62.06)	−113.94, 129.34	17.05 (62.06)	−104.59, 138.69	4.03 (62.72)	−118.90, 126.97	81.57 (79.84)	−74.91, 238.06
Birth length (cm)	0.05 (0.35)	−0.64, 0.74	0.11 (0.35)	−0.58, 0.80	0.16 (0.37)	−0.55, 0.89	0.63 (0.047)	−0.29, 1.55
Birth head circumference (cm)	−0.20 (0.18)	−0.55, 0.15	0.22 (0.18)	−0.13, 0.58	−0.18 (0.19)	−0.55, 0.19	0.46 (0.24)	−0.01, 0.94
Western dietary pattern								
Gestational weight gain (Kg)	−0.55 (0.74)	−2.002, 0.90	1.48 (0.74)	0.02, 2.93*	−0.55 (0.74)	−2.008, 0.89	1.19 (0.79)	−0.35, 2.75
Birth weight (g)	−28.55 (62.04)	−150.15, 93.05	−30.40 (62.04)	−152.00, 91.20	−35.49 (60.12)	−153.33, 82.34	−1.92 (35.64)	−128.05, 124.20
Birth length (cm)	−0.31 (0.35)	−1.004, 0.38	−0.71 (0.35)	−1.40, −0.02*	−0.32 (0.35)	−1.02, 0.36	−0.68 (0.37)	−1.42, 0.06
Birth head circumference (cm)	0.05 (0.18)	−0.31, 0.41	−0.07 (0.18)	−0.43, 0.29	0.05 (0.18)	−0.31, 0.41	−0.04 (0.19)	−0.43, 0.34
Healthy dietary pattern								
Gestational weight gain (Kg)	0.43 (0.75)	−1.04, 1.90	0.02 (0.75)	−1.45, 1.49	0.46 (0.74)	−1.001, 1.92	−0.14 (0.77)	−1.66, 1.38
Birth weight (g)	25.60 (61.94)	−95.80, 147.00	68.75 (61.94)	−52.65, 190.15	6.83 (60.08)	−110.94, 124.60	51.33 (62.64)	−71.44, 174.12
Birth length (cm)	−0.03 (0.35)	−0.72, 0.66	0.56 (0.35)	−0.12, 1.25	−0.08 (0.35)	−0.78, 0.60	0.54 (0.36)	−0.18, 1.26
Birth head circumference (cm)	−0.34 (0.18)	−0.70, 0.01	−0.09 (0.18)	−0.45, 0.26	−0.36 (0.18)	−0.72, 0.002	−0.10 (0.19)	−0.48, 0.26

*Note*: *p* Value is based on GLM. Tertile 1 is selected as *reference* group.

Abbreviations: CI: confidence interval; GLM, general linear model; SE, standard error of mean.

^a^ Adjusted for maternal age, maternal education, prepregnancy BMI, physical activity, and total energy intake.

* *p* < .05.

## 4 DISCUSSION

In the present study, the association between dietary patterns of pregnant women and pregnancy outcomes was examined for the first time in Guilan, the North of Iran. The results showed that pregnant women consuming the traditional pattern had more energy and macronutrient intake. In addition, women who consumed Western pattern had more GWG and reduced birth length only in the crude model.

The presence of specific food items in the content of our traditional pattern (characterized by high‐energy food items like sweets, bread and grains, processed meats, butter, soft drink, and high‐fat dairy) is justifiable for the higher energy and macronutrients in this category. Traditional pattern, as its name depicts, is specific to each country and region (Rodríguez‐Monforte et al., 2019). It should be mentioned that Iranian common diet contains high amounts of carbohydrates, especially refined bread, and grains. Moreover, consumption of fruits, vegetables, and low‐fat dairy products is low in Iran (Valipour et al., 2017). Therefore, this can be the reason why we observed significant association between traditional dietary pattern and higher energy and macronutrient intake.

The results about dietary patterns and pregnancy outcomes showed that high adherence to the Western pattern had a direct association with GWG and a reverse association with birth length before considering covariates. However, the results did not remain significant after considering covariates including maternal age, maternal education, prepregnancy BMI, physical activity, and total energy intake. It seems that the above‐mentioned factors have a strong confounding influence on the association between dietary patterns and pregnancy outcomes. In other words, though Western pattern was significantly associated with pregnancy outcomes, several other factors could strongly impact the results. Similar to our findings, in Cano‐Ibáñez et al. study, as an example, the association of Western diet and GWG was lost after adjustment for age of parity, social class, Kessner index, and smoking habits (Cano‐Ibáñez et al., 2020). However, Maugeri et al. reported a direct association between adherence to Western diet (characterized by high intakes of red meat, fries, dipping sauces, and salty snacks) and increased GWG (Maugeri et al., 2019). Itani et al. also found that Western pattern (characterized by high intakes of sugar‐sweetened beverages, fast food, and offal) was associated with excessive GWG rate (Itani et al., 2020). Several other studies (Angali et al., 2020; Ferreira et al., 2022; Tielemans et al., 2015; Uusitalo et al., 2009; Wrottesley et al., 2017) also indicated that high consumption of unhealthy diet had positive association with GWG. This means that besides dietary patterns (Hillesund et al., 2014; Shin et al., 2016), there were some other strong confounders such as sociodemographic and heath factors that could influence the relationships between dietary patterns and GWG among our pregnant population. However, we did not measure socioeconomic and health status of the study participants. For instance, with growing family income, pregnant women prefer to have healthy food, but with lower income, they consume unhealthier food (Angali et al., 2020). As for GWG, our significant reverse association between Western pattern and birth length was lost after adjustment. This is inconsistent with the findings of a cohort study which indicated a significant association between unhealthy dietary pattern and lower birth length (Mikeš et al., 2022). Another study also showed that high adherence to fat/sugar/takeaway pattern was associated with reduced birth length (Grieger et al., 2014).

Studies on dietary patterns and birth weight are in contradiction. The observation of no relationship between food patterns and baby's weight in the present study is in line with a cohort study of Coelho et al. with 1298 pregnant women, who showed no association between Western, traditional, and healthy patterns and birth weight (Coelho et al., 2015). However, another study on three patterns named Western, traditional, and healthy found lower odds of low birth weight with healthy and traditional patterns, but no association with Western pattern (Quansah & Boateng, 2020). Also, a cohort study in Brazil demonstrated reduced birth weight in women who had “meat, eggs, fried snacks, and processed food” dietary pattern or “sugars and sweets” dietary pattern (da Mota Santana et al., 2021). In the present study, we did not find any association between dietary patterns and head circumference and it is consistent with other studies (Hajianfar et al., 2018; Mikeš et al., 2022). In addition to maternal nutrition (Wrottesley et al., 2016), placenta structure (Sferruzzi‐Perri et al., 2017), and genetic and environmental factors could affect the growth of the fetus (Chen et al., 2018). Such reasons may justify why we did not observe a significant association between dietary patterns with birth weight, birth length, and head circumference.

Dietary patterns are strongly affected by a population's culture, geography, and regional condition in each area and can affect health outcomes (Englund‐Ögge et al., 2019; Hu, 2002; Kant et al., 2004). Therefore, the meaning of “Healthy” and “Unhealthy” is different between populations and makes it difficult to compare (Englund‐Ögge et al., 2019). Though the comparison of the results of different studies from one population to another is challenging, it is important, because this may help to understand and interpret the findings (Englund‐Ögge et al., 2019).

The present study had some limitations. First, it was a cross‐sectional study which could not show the causation. Memory problems to recall average portion sizes of foods through the FFQ is another limitation. Prepregnancy weight was derived from medical records or it was self‐reported; though it has been proved that self‐reported prepregnancy weight correlates well with measured prepregnancy weight (Harris & Ellison, 1998; Oken et al., 2007). Finally, we did not consider some other confounders such as economic status. Meanwhile, we examined food intake of pregnant mothers for the last trimester of pregnancy, which has the advantage of better recall than other trimesters. In addition, mothers' weight was measured just before delivery. We also used PCA approach for dietary patterns analysis which considers the correlation of food groups rather than focusing on selected aspects of a diet (Hoffmann et al., 2004). Furthermore, only a trained health researcher filled‐in the FFQ which increases the validity of data. We also adjusted the potential important confounders like age of mother, gestational age, prepregnancy BMI, physical activity, and energy intake. A unique strength of our study is that we excluded those who had changed their diet or had psychological problems during pregnancy which could influence GWG and birth outcomes.

## 5 CONCLUSIONS

The findings of the present study indicated that higher adherence to the Western diet consisting of high‐energy and unhealthy foods could result in greater GWG and lower birth length without considering the role of confounders. However, several other factors could strongly reverse the results. Therefore, the findings may be used to develop policies for interventional programs on fetal growth through not only improving maternal diet quality but also making lifestyle modifications to enhance their physical activity and pay special care to modifiable factors like prepregnancy weight among Iranian pregnant women.

## AUTHOR CONTRIBUTIONS

**Sahar Ghorbani‐Kafteroodi:** Conceptualization (equal); data curation (lead); formal analysis (equal); funding acquisition (equal); validation (equal); visualization (equal); writing – original draft (equal); writing – review and editing (equal). **Maryam Ghiasvand:** Formal analysis (equal); investigation (equal); resources (lead); software (lead); supervision (equal); validation (equal); visualization (equal); writing – original draft (equal); writing – review and editing (equal). **Maryam Saghafi‐Asl:** Conceptualization (lead); data curation (supporting); formal analysis (equal); funding acquisition (equal); investigation (equal); methodology (lead); project administration (equal); supervision (equal); validation (lead); writing – original draft (supporting); writing – review and editing (equal). **Soudabeh Kazemi Aski:** Data curation (supporting); project administration (supporting); supervision (supporting).

## CONFLICT OF INTEREST STATEMENT

The authors declare that there is no conflict of interest.

## ETHICS STATEMENT

The study was approved by the ethics committee of Tabriz University of Medical Sciences, Tabriz, Iran (Ethical code: TBZMED.REC.1400.220).

## PATIENT CONSENT STATEMENT

Written informed consent was obtained from all study participants prior to their inclusion in the study. Patient anonymity has been preserved.

## Data Availability

Our data have not been shared anywhere in any thesis or article and is original. We confirm the presence of shared data. If needed, the data can be accessed from the author.
